# The crossroad between autoimmune disorder, tissue remodeling and cancer of the thyroid: The long pentraxin 3 (PTX3)

**DOI:** 10.3389/fendo.2023.1146017

**Published:** 2023-03-21

**Authors:** Damiano Chiari, Barbara Pirali, Vittoria Perano, Roberto Leone, Alberto Mantovani, Barbara Bottazzi

**Affiliations:** ^1^ Department of Biomedical Sciences, Humanitas University, Pieve Emanuele, Italy; ^2^ General Surgery Department, Humanitas Mater Domini Clinical Institute, Castellanza, Italy; ^3^ Endocrinology Clinic, Internal Medicine Department, Humanitas Mater Domini Clinical Institute, Castellanza, Italy; ^4^ IRCCS Humanitas Research Hospital, Rozzano, Italy; ^5^ Harvey Research Institute, Queen Mary University of London Charterhouse Square, London, United Kingdom

**Keywords:** PTX3, inflammation, thyroid disorders, thyroid cancer, nodular thyroid disease, Graves' disease

## Abstract

Thyroid is at the crossroads of immune dysregulation, tissue remodeling and oncogenesis. Autoimmune disorders, nodular disease and cancer of the thyroid affect a large amount of general population, mainly women. We wondered if there could be a common factor behind three processes (immune dysregulation, tissue remodeling and oncogenesis) that frequently affect, sometimes coexisting, the thyroid gland. The long pentraxin 3 (PTX3) is an essential component of the humoral arm of the innate immune system acting as soluble pattern recognition molecule. The protein is found expressed in a variety of cell types during tissue injury and stress. In addition, PTX3 is produced by neutrophils during maturation in the bone-marrow and is stored in lactoferrin-granules. PTX3 is a regulator of the complement cascade and orchestrates tissue remodeling and repair. Preclinical data and studies in human tumors indicate that PTX3 can act both as an extrinsic oncosuppressor by modulating complement-dependent tumor-promoting inflammation, or as a tumor-promoter molecule, regulating cell invasion and proliferation and epithelial to mesenchymal transition, thus suggesting that this molecule may have different functions on carcinogenesis. The involvement of PTX3 in the regulation of immune responses, tissue remodeling and oncosuppressive processes led us to explore its potential role in the development of thyroid disorders. In this review, we aimed to highlight what is known, at the state of the art, regarding the connection between the long pentraxin 3 and the main thyroid diseases i.e., nodular thyroid disease, thyroid cancer and autoimmune thyroid disorders.

## 1 Background

Thyroid gland is at the crossroads of immune dysregulation, tissue remodeling and oncogenesis (1). Thyroid problems are among the most widespread illness affecting the global population: autoimmune disorders, nodular disease and cancer of the thyroid affect 2 - 5% of the population, with higher incidence in women, and are the most prevalent organ-specific autoimmune diseases (2). The prevalence of thyroid nodules detected with ultrasound is 19-35% (3). In the world the incidence rates of new cases of thyroid cancer were about 10·1 and 3·1 per 100.000 women and men, respectively (4). Many people are affected by mild thyroid dysfunction and only a small part of these have a severe evolution. The identification of novel tools for the diagnosis, screening and monitoring of patients affected by thyroid illness could improve patient management by reducing unnecessary diagnostic tests and interventions.

The long pentraxin 3 (PTX3) is a soluble pattern recognition molecule and an essential mediator of the innate immune response, expressed by a variety of cell types of hematopoietic or stromal origin (i.e. macrophages, endothelial cells, fibroblasts, etc.) during tissue injury and stress or upon stimulation with pro-inflammatory signals (5–7). PTX3 is also produced by neutrophils during the maturation process in the bone marrow and it is stored in specific granules (8). The molecule exerts a role in tissue repair and remodeling, and acts as regulator of complement activation and leukocytes recruitment (9–15). In addition, PTX3 levels rapidly increase during inflammatory conditions (infectious diseases, sepsis, acute respiratory distress syndrome, cardiovascular diseases), correlating with severity and predicting the risk of mortality (16–21). Increased levels of PTX3 were also observed in different autoimmune disorders, such as small-vessel vasculitis and rheumatoid arthritis (22–27).

Several tumors express PTX3, including lung cancer, glioma, ovarian cancer, myxoid liposarcoma, prostate carcinoma, esophageal squamous cell carcinoma and pancreatic cancer (28–32). The role of PTX3 in tumorigenesis is still poorly defined (33, 34). In preclinical models, *Ptx3* deficiency was associated to higher susceptibility to chemical carcinogenesis (9), while in human prostate cancer PTX3 expression is progressively reduced in high-grade prostatic intraepithelial neoplasia and invasive tumor areas (35). In addition, epigenetic studies showed promoter hypermethilation and silencing of PTX3 expression in selected human tumors, such as colorectal cancer and esophageal squamous cell carcinoma (9, 30, 36–38). However, in other contexts (i.e. glioma, head and neck cancers, hepatocellular carcinoma) PTX3 overexpression has a pro-tumorigenic effect, promoting cell invasion or proliferation and epithelial to mesenchymal transition (39–43). PTX3 is therefore playing a complex and still undefined role in the regulation of immune response, tissue remodeling and oncosuppressive processes.

In this review we will explore the role of PTX3 in the development of thyroid diseases, analyzing studies including patients with Graves’ disease (GD), thyroid nodules and thyroid cancer supported also by results obtained by our group.

## 2 The link between PTX3 and thyroid diseases

### 2.1 Benign thyroid nodules

The nodular development of the thyroid results from multifocal monoclonal or polyclonal proliferation of thyrocytes producing new follicles or structures similar to follicles considerably heterogeneous from functional and morphological points of view (44). More than 90% of detected nodules in adults are noncancerous, and thyroid stimulating hormone (TSH) is the main mitotic factor in nodule formation (45). Radioactive sources, many habits (iodine intake, smoking, etc.), harmful chemicals, metabolic syndrome and genetic mutations may cause chronic inflammation, contributing to nodule and cancer formation (46, 47). Destek et al. investigated the relationship of some inflammatory and autoimmune markers, among which PTX3, with thyroid nodules characteristics (48). They did not observe differences of PTX3 plasmatic levels between nodular goiter group and control group, nor correlations between PTX3 levels and nodule characteristics.

**Figure 1:**
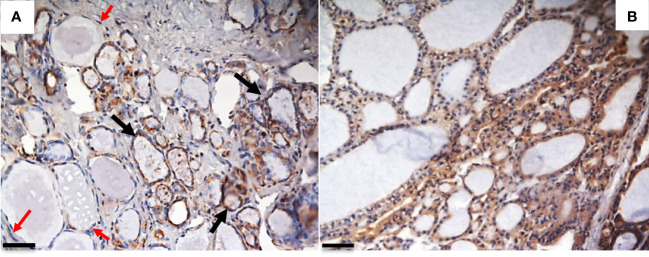
PTX3 immunolocalization in tissue samples of remodeling thyroid tissue. Goiter and GD specimens were obtained after surgical removal of the thyroid, frozen in liquid N2-cooled isopentane, and stored at -80°C until immunohistochemical analysis. Sections were stained with rabbit anti-human PTX3 and analyzed with Nikon Eclipse 55i microscope. Images were captured with a Digital Sight DS-5 M digital camera (Nikon) using Lucia G software (Laboratory Imaging). **(A)** Goiter: PTX3 staining (brown) is evident in epithelial follicular cells of some thyroid follicles (black arrow), while some follicles do not express PTX3 (red arrows); **(B)** GD: a diffuse PTX3 positive staining is evident for all the follicles (immunoperoxidase staining; magnification: **(A)** 20x; **(B)** 20x; scale bar, 100μm). The analysis was conducted in accordance with local ethical guidelines. The patient has provided informed clinical consent.

**Figure 2:**
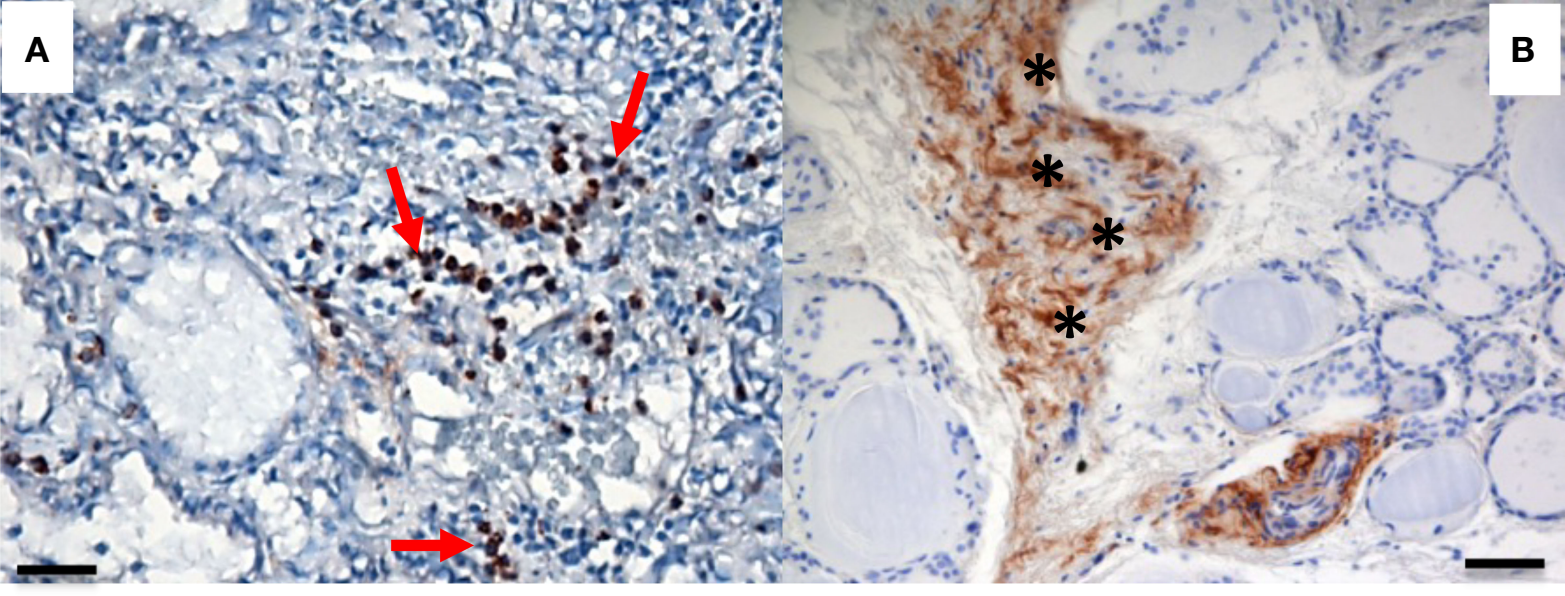
PTX3 immunolocalization in tissue samples of remodeling thyroid tissue. PTX3 immunostaining (brown) of thyroid tissue sections was performed as in **Figure 1**. **(A)** GD: PTX3 immunostaining of immune cells infiltrating the parenchima (red arrow). **(B)** Goiter: PTX3 immunostaining at the level of the extracellular matrix (asterisk). (immunoperoxidase staining; magnification: **(A)** 20x; **(B)** 20x; scale bar, 100μm).

We recently evaluated the plasmatic levels of PTX3 in a group of 53 patients undergoing thyroidectomy for both benign and malignant nodules. A preliminary analysis revealed that preoperative plasmatic PTX3 levels were significantly higher than normal in patients with thyroid disease (p<0.05). Plasmatic PTX3 mean value was 4.54 ng/ml (range 1.06 – 8.63 ng/ml), when normal value is considered 2 ng/ml with 1 ng/ml of standard deviation. Preoperative PTX3 levels were not significantly different between benign and malignant nodules. At 45 days follow-up PTX3 mean value was reduce to 3.40 ng/ml (range 0.89 – 9.21 ng/ml); this reduction was statistically significant (p<0.05) (49). Moreover, we observed PTX3 expression in thyroid tissue by immune infiltrate, extracellular matrix and follicular cells (**Figures 1**, **2**).

### 2.2 Thyroid carcinoma

It is well known that PTX3 modulates the cancer-related inflammation or angiogenesis involved in the carcinogenetic process of several types of cancer (33, 34). Other studies support the hypothesis that PTX3 exerts a pro-tumorigenic effect promoting macrophage infiltration and tumor cell migration and invasion. It is possible that PTX3 may have different functions on cancer development depending on the tissue and cancer type (33). The role of PTX3 as biomarker of cancer has been investigated. In patients with lung cancer, prostate cancer or colorectal carcinoma, blood PTX3 levels resulted elevated compared respectively to healthy subjects, patients with prostatic inflammation or colorectal polyps (50–52).

Ninety to 95% of thyroid cancers are categorized as well-differentiated tumors arising from the follicular cells. Papillary and follicular carcinomas are included in this category. Papillary carcinoma is the most common of the thyroid neoplasms (53) and is usually associated with an excellent prognosis. Follicular carcinoma is the second category of well-differentiated thyroid cancer and is a disease of older population. Anaplastic thyroid carcinoma (ATC) represents less than 1% of thyroid malignancies and it is the most aggressive form of thyroid cancers. Medullary thyroid carcinoma arises from the parafollicular cells (or C cells) and accounts for 5% to 10% of thyroid cancers.

A recent study identified a four genes signature including PTX3, 3′-phosphoadenosine 5′-phosphosulfate synthase 2 (PAPSS2), procollagen C-endopeptidase enhancer 2 (PCOLCE2) and transforming growth factor beta receptor 3 (TGFBR3) for papillary carcinoma as marker for risk stratification and survival prediction (54). PTX3 expression was significantly lower in patients surviving with tumors than in tumor free patients, revealing a potential correlation between PTX3 and tumor recurrence.

Another study found that PTX3, Collectin Subfamily Member 12 (COLEC12) and Platelet Derived Growth Factor receptor alfa (PDGFRA) could be used as biomarkers to differentiate the anaplastic thyroid carcinoma from follicular or papillary thyroid carcinomas (55). These three genes are overexpressed in ATC meanwhile under-expressed in the other two subtypes.

### 2.3 Graves’ disease

In recent years, growing numbers of studies have indicated that abnormal expression of PTX3 may play a role in the pathogenesis and development of several autoimmune diseases (rheumatoid arthritis, systemic lupus erythematosus, etc.) (56). Hashimoto’s thyroiditis is the most common cause of hypothyroidism in iodine-sufficient areas. It is characterized clinically by gradual thyroid failure, due to lymphocytic infiltration and autoimmune-mediated destruction of the thyroid. Extremely scarce data can be found in literature regarding the relationship between this disorder and PTX3. An abstract reported that there is not a significant difference in PTX3 levels between patients with autoimmune thyroiditis and control group (57). Possibly, the lack of PTX3 overexpression in Hashimoto’s thyroiditis could be due to the inability of lymphocytes, mainly involved in this pathology, to produce PTX3, neither constitutively nor after appropriate stimulation (7). Therefore, we focused on Graves’ disease and PTX3. GD is an organ-specific autoimmune disease in which thyroid-stimulating immunoglobulins (TSI) activate the TSH receptor (TSHR) expressed on thyroid epithelial cells causing an overproduction of thyroid hormones (58). As a consequence, GD typically causes hyperthyroidism. Other possible clinical manifestations of GD are thyroid enlargement, protruding eyes, and pretibial myxedema. Cheng et al. showed that PTX3 levels are higher in GD (59) and we observed that PTX3 is expressed in thyroid epithelial follicular cells in a cohort of 14 patients that underwent total thyroidectomy for GD (**Figure 1B**). GD frequently manifests with a thyroid-associated ophthalmopathy (TAO), characterized by inflammation and remodeling of orbital connective tissues and extraocular muscles (60). After a symptomatic active phase, the inflammation and congestion signs may alleviate, and the disease gradually transits to the inactive phase (chronic fibrosis). As observed by Zhang et al. (61), PTX3 can form a complex with hyaluronan and it is possible that is involved in the tissue remodeling process in case of TAO. Expression of PTX3 in orbital connective tissues in active TAO was reported in earlier publications (62). Orbital fibrocytes are the leading actor of TAO and seem to derive from CD34+ fibrocytes (bone marrow–derived monocyte progenitor cells) (63). The expression of functional TSHR and other “thyroid-specific” proteins is a common characteristic between fibrocytes and CD34+ orbital fibroblast. Fibrocytes express several cytokines and other inflammatory genes, including PTX3, when activated through TSHR. According to Wang et al., at a pre-translational level TSH induces PTX3 expression by orbital fibroblasts; in addition, basal levels of PTX3 in GD orbital fibroblasts seem to be higher than those in healthy-orbital fibroblasts (64). Mou et al. (65) demonstrated higher PTX3 mRNA expression in the orbital adipose-connective tissue from TAO patients compared to healthy subjects. They also observed higher serum PTX3 concentration in patients compared to the control group, but not among active and inactive TAO. Other authors observed that serum levels of PTX3 and other cytokines were higher in active GD and that were associated with thyroid-stimulating hormone receptor antibodies (TRAbs) levels (59). These findings diverge from Diao et al. (66) who observed that basal levels of PTX3 mRNA in TAO-orbital fibroblasts did not seem to be different from those in healthy-orbital fibroblasts, but they had enrolled only patients with stable and inactive TAO. The current literature suggests that PTX3 could be a potential therapeutic molecule of an antifibrotic treatment (5). Diao et al. demonstrated *in vitro* that TGF-β1 inhibits PTX3 expression in human orbital fibroblasts with TAO (66). In addition, dexamethasone and IGF-1 receptor–blocking antibodies (teprotumumab and 1H7) attenuate the TSH-mediated induction of PTX3 *in vitro* (64).

## 3 Conclusions

Thyroid gland is at the crossroads of immune dysregulation, tissue remodeling and oncogenesis. We hypothesized the existence of a common mechanism underlying autoimmune disorders, nodular disease and cancer of the thyroid. PTX3 is a key component of the humoral arm of innate immunity regulating immune responses, tissue remodeling and oncosuppressive processes, all potentially involved in the development of thyroid diseases.

From histological point of view, PTX3 resulted expressed in the thyroid gland by different cells: infiltrating immune cells, extracellular matrix and follicular cells. Different patterns of expression could represent different mechanism of actions of PTX3 in the etiopathogenesis of thyroid disorders.

Chronic inflammation is considered a possible cause of nodule development. Destek et al. showed that plasmatic PTX3 levels were not different between patients with thyroid nodules compared to controls (48). On the other side, we observed significantly higher plasmatic levels of PTX3 in a cohort of patients who underwent thyroidectomy for nodular goiter (49). The indication to surgery reflects a situation of remodeling of the gland in terms of dimensional increase or hyperfunction. Therefore, we can hypothesize that PTX3 overexpression is associated with active phases of nodular remodeling.

Thyroid carcinoma, with the four subtypes papillary follicular, anaplastic and medullary, is the most common endocrine malignancy. Two different gene signatures including PTX3 were identified for the subtypes arising from the follicular cells: papillary, follicular and anaplastic. These new evidences suggest that the molecule could be an independent prognostic factor for most of the thyroid carcinomas (54), and a diagnostic tool to differentiate the anaplastic thyroid carcinoma from the other two subtypes (55).

PTX3 is also involved in GD. Several authors observed higher PTX3 levels in the orbital fibroblasts of patients with TAO. Published data suggest that PTX3 is overexpressed during the active phases of the disease and it returns to baseline levels once TAO reaches a stable phase (59, 61, 62, 64, 65).

Overall, the data summarized here suggest that PTX3 could play a role both in thyroid cancer, as demonstrated by the presence of PTX3 among the gene signatures associated to the different subtypes of thyroid carcinomas, and in the nodular remodeling of the thyroid, as suggested by PTX3 overexpression from goiter patients and GD patients. Ongoing and future researches are required to better understand these aspects and, from a clinical point of view, to determine the possible role of PTX3 as a biomarker of thyroid cancer and as a possible therapeutic target in GD.

## Author contributions

DC conceived the study; BP, VP and DC were the major contributors in writing the manuscript; RL was involved in experimental activities; AM and BB were involved in drafting the manuscript and revising it critically. All authors contributed to the article and approved the submitted version.
